# Vitamins K1 and K2: The Emerging Group of Vitamins Required for Human Health

**DOI:** 10.1155/2017/6254836

**Published:** 2017-06-18

**Authors:** Gerry Kurt Schwalfenberg

**Affiliations:** Department of Family Medicine, University of Alberta, No. 301, 9509-156 Street, Edmonton, AB, Canada T5P 4J5

## Abstract

**Objective:**

To review the evidence for the use of vitamin K supplementation in clinical conditions such as osteoporosis, vascular calcification, arthritis, cancer, renal calculi, diabetes, and warfarin therapy.

**Quality of Evidence:**

PubMed was searched for articles on vitamin K (K1 and K2) along with books and conference proceedings and health conditions listed above. Level I and II evidence supports the use of vitamins K1 and K2 in osteoporosis and Level II evidence supports vitamin K2 in prevention of coronary calcification and cardiovascular disease. Evidence is insufficient for use in diabetes, arthritis, renal calculi, and cancer.

**Main Message:**

Vitamin K2 may be a useful adjunct for the treatment of osteoporosis, along with vitamin D and calcium, rivaling bisphosphonate therapy without toxicity. It may also significantly reduce morbidity and mortality in cardiovascular health by reducing vascular calcification. Vitamin K2 appears promising in the areas of diabetes, cancer, and osteoarthritis. Vitamin K use in warfarin therapy is safe and may improve INR control, although a dosage adjustment is required.

**Conclusion:**

Vitamin K supplementation may be useful for a number of chronic conditions that are afflicting North Americans as the population ages. Supplementation may be required for bone and cardiovascular health.

## 1. Introduction

Vitamin K is a name given to a group of fat-soluble vitamins. They are considered essential cofactors in humans for the production of several proteins that are involved in coagulation homeostasis and calcium homeostasis. The original term vitamin “K” comes from the K in the Germanic word Koagulation meaning the ability to clot blood or prevent hemorrhage. Much has been learned about vitamin K2 and its role in osteoporosis, vascular calcification, osteoarthritis, cancer, and cognition over the past few years. The most commonly known vitamin K types are listed in Table 1, along with their corresponding functions and sources.

Deficiency of vitamin K2 has been linked with vascular calcification and osteoporosis [1]. Matrix GLa protein (MGP) is a vitamin K-dependent protein that inhibits vascular and soft tissue calcification when activated.

Vitamin K is also a cofactor for carboxylation of glutamate to gamma carboxyglutamic acid (GLa). GLa containing bone proteins are synthesized by osteoblasts and have been identified as osteocalcin, matrix GLa protein, and pit protein S. Carboxylated osteocalcin (OC) increases after vitamin K2 administration and there is a connection between uncarboxylated OC and the risk of clinical fractures [2]. Vitamin K2 (MK-4) supplementation is quite safe and does not induce hypercoagulation even at doses of 15 mg three times a day [3].

## 2. Quality of Evidence

For this paper a traditional integrated review format was chosen [4]. This approach seems more appropriate when incorporating and synthesizing the literature where there is limited primary study, while endeavoring to provide a useful overview of the literature to professionals in the medical community. Available medical and scientific literature from PubMed, MEDLINE, books, and conference proceedings was assessed. Terms searched were vitamin K (K1 and K2) and the role in osteoporosis, vascular calcification, osteoarthritis, diabetes, cancer, cognitive function, and interaction with warfarin and vitamin D. The American Family Physician toolkit for Evidence-Based Medicine level of evidence (LOE) was applied to studies in relation to the findings in each area as available.

## 3. Vitamin K Deficiency

The daily recommended requirement for vitamin K is 90 *μ*gm/day for women and 120 *μ*gm/day for men [8]. Sources of vitamins K1 and K2 are listed in Table 2. Deficiency based on bleeding problems is rare, except in newborns. Prior to the use of prophylactic vitamin K injections in neonates, deficiency of vitamin K would result in a hemorrhagic condition with associated cutaneous, intrathoracic, gastrointestinal, and intracranial bleeding.

A more sensitive indicator of vitamin K deficiency would be a measure of uncarboxylated osteocalcin or uncarboxylated GLa proteins. Undercarboxylated osteocalcin is considered a marker for hip fracture risk [9]. This may be more relevant now that we understand the function of vitamin K2 in the vascular system and bone health.

There are a number of conditions and medications that interfere with vitamin K absorption, which are listed as follows:

*Vitamin K Interactions and Vitamin K Absorption*

- Antibiotic use (longer than 10 days)
- Dilantin (use in pregnancy or nursing may deplete vitamin K in newborns)
- Low fat diet and fat blocking supplements
- Bile acid sequestrants (which prevent fat absorption) such as cholestyramine, colestipol, or colesevelam
- Orlistat, Xenical, and Olestra (FDA requires addition of vitamins K, A E, and D to food products containing Olestra)
- Mineral oil
- Preservative butylated hydroxytoluene (BHT)
- GI tract diseases, liver diseases, and estrogen drugs

Source: http://umm.edu/health/medical/altmed/supplement-interaction/possible-interactions-with-vitamin-k.

## 4. Vitamin K and Osteoporosis

Vitamin K2 appears to improve bone quality, which leads to a reduction in fractures; however, bone density may not always be affected in some studies. The lifetime risk of having at least one fracture is reduced by 25% with the daily use of 800 IU vitamin D, 45 *μ*gm vitamin K2, and 1200 mg calcium [10]. Vitamin K2 (MK-7) from fermented soybeans stimulates osteoblasts and inhibits osteoclasts resulting in an anabolic effect on bone calcification [11]. A systematic review (level of evidence I [LOE = A]) has shown vitamin K2 to prevent fractures in vertebra by 60%, hip fractures by 77%, and nonvertebral fractures by 81% in Japanese patients [12]. This rivals conventional bisphosphonate therapy. A study (LOE-B) with 241 osteoporotic patients treated with vitamin K2 (45 *μ*gm/day) along with calcium showed that they maintained their bone density, whereas those on calcium and placebo lost 2.5% of their lumbar bone density. Furthermore, the treatment group had 65% fewer fractures [13]. In clinical studies, vitamin K2 maintains lumbar bone mineral density (BMD), reduces age-related osteoporotic fractures, reduces glucocorticoid-induced osteoporotic vertebral fractures, and maintains lumbar BMD in liver-dysfunction-induced osteoporosis and in paralytics it increases the metacarpal BMD in upper extremities of patients with cerebrovascular disease [14]. A three-year randomized control trial (RCT) (LOE = A) study showed that supplementing vitamin K2 at 180 *μ*gm/day reduced the usual age-related decline in BMD in the lumbar spine and femoral neck but not the total hip. Vitamin K2 (MK-7) also prevented the loss in vertebral height in the lower thoracic spine [15].

Supplementation of low dose vitamin K1 (500 *μ*gm/day) for 3 years (LOE-B) did not improve bone density in the treatment group [16]. Another study where vitamin K1 was used for two years resulted in no significant change in bone density compared to placebo. However, there were significantly fewer fractures in the treatment group (50% reduction) [17]. Also noted was a significant reduction of incident cancers in the treatment group (LOE = A).

The United States and Canada do not have recommendations for the use of vitamin K1 for osteoporosis as well as no recommendations for vitamin K2. Vitamin K2 is recommended as standard of care in Japan where most of these studies have taken place.

Vitamin D, calcium, and vitamin K2 supplementation reduces undercarboxylated osteocalcin and improves lumbar bone mineral density [18]. Thus, the addition of vitamin K2 is essential for good bone health.

## 5. Vitamin K and Cardiovascular Disease

Vitamin K inhibits vascular calcification by matrix GLa proteins. These proteins are activated via vitamin-dependent carboxylation. Activated matrix GLa protein identified in atherosclerotic plaque may prevent calcium precipitation [19] and soft tissue calcification [20]. In a prospective population-based study (LOE-A) of 4807 subjects free from myocardial infarction at baseline followed up for 7 years, the odds ratio of the highest tertile intake of menaquinone (vitamin K2) compared to the lowest resulted in a significant risk reduction in coronary heart disease, 0.43 (CI 0.34–0.77); all-cause mortality, 0.74 (CI 0.59–0.92); and severe aortic calcification, 0.48 (CI 0.32–0.71). The intake of phylloquinone (vitamin K1) was not found to impact any of the targeted outcomes [19]. A cohort study (LOE = B) of 16057 women free from cardiovascular disease at baseline with a mean follow-up of 8.1 years revealed that for every 10 *μ*gm increase in vitamin K2 intake there was a 9% reduction in coronary events. Again, vitamin K1 intake was not significantly related to cardiovascular outcomes [21]. One study found that low serum vitamin K1 in antihypertensive medication users was significantly associated with coronary artery calcium progression [22].

## 6. Vitamin K and Arthritis 

Emerging data is revealing that vitamin K may be important in preventing disabling osteoarthritis. Abnormal mineralization of cartilage and bone has been seen with insufficient vitamin K intake [23]. A longitudinal study comparing patients who have subclinical vitamin K deficiency to those that have adequate intake has shown an increased risk of developing knee osteoarthritis (risk ratio [RR]: 1.56; 95% confidence interval [CI], 1.08–2.25) and cartilage lesions (RR: 2.39; 95% CI, 1.05–5.40) [24]. An 3-year RCT (LOE = A) assessing vitamin K1 supplementation versus placebo showed no overall effect of vitamin K on radiographic hand arthritis; however, those who had insufficient vitamin K at baseline that later attained sufficient concentration at follow-up did have a trend to less joint space narrowing (47% less joint space narrowing) [25].

There is evidence that vitamin K2 supplementation reduces inflammation in rheumatoid arthritis by reducing CRP levels [26]. Vitamin K2 may induce apoptosis in rheumatoid arthritis synovial cells. In a cross-sectional study (LOE = B), the group given 100 *μ*gm of MK-7 had a significant reduction in disease activity score along with improved biochemical markers (ESR, CRP, and matrix metalloproteinase) after 3 months [27].

## 7. Vitamin K and Renal Calculi

Urinary GLa protein inhibits precipitation of various calcium salts. Vitamin K is required for the carboxylation and activation of this protein [28]. It has been suggested that reduced carboxylase activity such as that seen in urolithic patients may play an important role in calcium oxalate urolithiasis [29].

## 8. Vitamin K and Diabetes

Even though it is known that there are high levels of vitamin K in the pancreas, deficiency in vitamin K results in excessive insulin release and reduces clearance of glucose from the blood in rats [30]. Recently, a placebo controlled trial (LOE = A) showed that using 30 mg of vitamin K2 supplementation increased insulin sensitivity in healthy young men via osteocalcin metabolism [31]. Vitamin K1 500 *μ*gm/day for 36 months improved insulin resistance (significantly lower HOMA-IR) in men but not in women [32]. Increased vitamin K1 intake in a cohort study (LOE = B) was shown to decrease risk of developing diabetes by 51%. A recent review suggests that vitamin K supplementation may be used as a novel adjuvant therapy to improve glycemic control and quality of life [33].

## 9. Vitamin K and Cancer

Much research is taking place presently looking at the vitamin K family and its potential anticancer effect [34]. Vitamin K2 may safely suppress growth and invasion of human hepatocellular carcinoma via protein kinase A activation and result in moderate suppression of tumor recurrence [35]. It has also been shown to result in growth suppression in a dose dependent manner in lung cancer cells in vitro [36]. Similar results were found in pancreatic cancer cells [37]. A cohort study (LOE = B) of over 11,000 patients showed that higher vitamin K2 intake was associated with a significant reduction in advanced prostate cancer in particular [38]. There was no association with higher vitamin K1 intake and reduction of prostate cancer.

## 10. Vitamin K and Cognition

The essential role of vitamin K in the synthesis of sphingolipids in the brain has been known for more than 40 years [39]. More recently, vitamin K dependent proteins such as Protein Gas6 have been shown to play a key role in the peripheral and central nervous system [40]. Vitamin K may have a role in the pathogenesis of Alzheimer's disease because of its regulatory role in sulfotransferase activity and growth factor/tyrosine kinase receptor activity in the brain [41]. There is evidence that vitamin K1 intake in the elderly with Alzheimer's disease is significantly lower than in controls in the community [42]. Intake of vitamin K may improve cognitive function in healthy older adults. One such study showed that vitamin K1 was associated with better verbal episodic memory performances especially on recall tasks [43]. The use of vitamin K antagonists has been associated with more frequent cognitive impairment [44].

## 11. Warfarin and Vitamin K Interactions

Warfarin anticoagulation results in osteoporosis and the need for vitamin K2 [45]. A study using vitamin K1 (150 *μ*gm phytomenadione) daily in patients with unstable anticoagulation control showed that increasing and stabilizing the body's stores of the vitamin allowed for better control of anticoagulation by maintaining steady activation of vitamin K-dependent clotting factors [46]. Recently, a study (LOE = A) has confirmed this again [47]. In the group receiving vitamin K supplementation, the median number of warfarin dosage changes was significantly lower than in the placebo group. The dose of warfarin required for the treatment group receiving 150 *μ*gm of vitamin K1 was 16% greater than the control group.

Considerations of vitamin K supplementation with anticoagulation should include dose and type of vitamin K used. Extended intake of vitamin K1 of 700 *μ*gm reduced INR values from 2 to 1.5. Vitamin K2 supplementation is more potent at reducing INR and 200 *μ*gm of K2 will reduce INR values from 2 to 1.5. Thus, supplementation of >50 *μ*gm of vitamin K2 requires INR monitoring [48].

The evidence that coumadin may increase fractures, arterial calcification, and mortality is still in conflict. One study looking at hemodialysis patients showed an increase risk of fractures in males but not in females. Also, there was a significant increase in aortic and iliac calcification. Alarmingly, the hazards ratio for all-cause mortality was 2.42 in the warfarin treated group [49]. A recent case control study (LOE = B) looking at warfarin use in men has shown an increase in advanced prostate cancer by 220% after more than 4 years of use [50]. In another study, long-term warfarin use and risk for fractures compared to a matched cohort did not reveal an increased risk of fractures [51].

## 12. Conclusion 

Some of the recent review articles suggest that there is insufficient information in the literature to recommend the use of vitamin K1 supplements to prevent bone loss, fractures, and osteoarthritis in humans [52]. Researches looking at these effects when supplementing vitamin K1 on bone density and vascular calcification are generally negative or show no difference.

Studies using vitamin K2 demonstrate improvement in bone quality rather than bone density, while significantly reducing fractures and preventing vascular calcification. For this reason, the literature is sometimes confusing and care must be taken to clearly look at the differences in actions of vitamins K1 and K2. There is a need for more research to be done on vitamin K2 in regard to its effect on arthritis, cognition, diabetes, renal calculi, and cancer.

Vitamin K2 in the form of MK-7 is rapidly becoming popular as a supplement and is available OTC usually with a dose of 100–120 *μ*gm. It is important as physicians to be aware that MK-7 can interfere with anticoagulation therapy when used above 50 *μ*gm/day [48]. On the other hand, the supplementation of some vitamin K at a steady level during anticoagulation therapy may result in a more stable INR that requires fewer adjustments. Using a small dose of vitamin K2 may benefit the patient by reducing the risk of osteoporosis, osteoarthritis, and vascular and tissue calcification. Well-controlled RCT studies are urgently needed in this area, especially given the well tolerated safety profile of vitamins K1 and K2.

Newer agents for anticoagulation such as dabigatran, rivaroxaban, and apixaban are not vitamin K-dependent. This would allow for the safer use of higher doses of vitamin K to prevent atherosclerosis, osteoporosis, and cognitive impairment, which may have the potential to reduce morbidity and mortality in this patient population [53].

The use of vitamin D and vitamin K2 together as an approach to osteoporosis treatment may significantly reduce morbidity and mortality. This approach may rival bisphosphonate treatment without the side effects associated with the use of this medication, along with reducing vascular calcification and its complications.

## Figures and Tables

**Table 1:** Vitamin K types, functions, and sources.

Type of vitamin K	Function in the human body	Sources of vitamin
Vitamin K1	(i) Participates in blood clotting. Serves as a cofactor for carboxylation of protein bound glutamate residues by converting them to carboxy glutamate (GLa). GLa containing proteins are found in Factors II, VII, IX, and X	(i) Green leafy vegetables and some plant oils

Vitamin K2, menaquinone-4 (MK-4)	(i) Osteocalcin (synthesized in bone) (ii) Matrix GLa protein (synthesized in cartilage and in blood vessel walls) It is involved in calcium transport, preventing calcium deposition in the lining of blood vessel walls, and helps improve bone density [1] (iii) Short chain form with shorter half-life	(i) Butter, eggs yolks, lard, and animal based foods (ii) Synthesis by bacteria in the intestinal tract (however, synthesized MK-4 is bound to the membranes of bacteria in the gut and very little is absorbed in humans) [5] (iii) Over-the-counter (OTC) supplements

Vitamin K2, menaquinone-7 (MK-7)	(i) As for MK-4 (ii) Long chain form with longer half-life	(i) Fermented foods, some cheese (ii) Extracted from Nattō (fermented soy) as an OTC supplement

Vitamin K3, menadione	(i) Has been banned by the FDA in the USA because of potential toxicity (hemolytic anemia) [6] (ii) Is presently being studied as a potential prostate/hepatocellular cancer therapy and potential treatment for skin toxicities secondary to kinase inhibitor therapy [7]	(i) ⁡Synthetic analogue of vitamin K considered a provitamin

**Table 2:** Food sources of vitamins K1 and K2.

Vitamin K1	Vitamin K2
(1) Boiled spinach	(1) Nattō (fermented soy)
(2) Cooked broccoli	(2) Hard cheese (Gouda)
(3) Coleslaw with homemade dressing	(3) Soft cheese (blue cheese)
(4) Cooked asparagus	(4) Egg yolk
(5) Soybean oil	(5) Butter
(6) Red or green grapes	(6) Chicken liver
(7) Plums	(7) Salami
(8) Kidney beans	(8) Chicken breast
(9) Yogurt	(9) Ground beef
(10) Mayonnaise	(10) Sauerkraut
(11) Margarine	(11) Fermented milk (kefir)

*Source*. USDA National Agricultural Library, Composition Vitamins and Minerals. Kamao M, S., et al, *Vitamin K Content of Foods and Dietary Vitamin K Intake in Japanese Young Women.* J Nutr Sci Vitaminol (Tokyo), 2007. 53: p. 464-470.

## References

[1] Flore R., Ponziani F. R., Di Rienzo T. A. (2013). Something more to say about calcium homeostasis: the role of vitamin K2 in vascular calcification and osteoporosis. *European Review for Medical and Pharmacological Sciences*.

[2] Vergnaud P., Garnero P., Meunier P. J., Bréart G., Kamihagi K., Delmas P. D. (1997). Undercarboxylated osteocalcin measured with a specific immunoassay predicts hip fracture in elderly women: the EPIDOS study. *Journal of Clinical Endocrinology and Metabolism*.

[3] Asakura H., Myou S., Ontachi Y. (2001). Vitamin K administration to elderly patients with osteoporosis induces no hemostatic activation, even in those with suspected vitamin K deficiency. *Osteoporosis International*.

[4] Dijkers M. P. J. M., Task Force on Systematic and Guidelines (2009). The value of traditional reviews in the era of systematic reviewing. *The American Journal of Physical Medicine and Rehabilitation*.

[5] Unden G., Bongaerts J. (1997). Alternative respiratory pathways of Escherichia coli: energetics and transcriptional regulation in response to electron acceptors. *Biochimica et Biophysica Acta (BBA) - Bioenergetics*.

[6] Vetrella M., Barthelmai W. (1972). Studies on drug-induced hemolysis: effects of menadione and its water soluble preparations on the glutathione peroxidase of human erythrocytes. *Klinische Wochenschrift*.

[7] Chen J., Jiang Z., Wang B., Wang Y., Hu X. (2012). Vitamin K(3) and K(5) are inhibitors of tumor pyruvate kinase M2. *Cancer Letters*.

[8] Otten J. J. H. J., Meyers L. D. (2008). *S Government Food and Nutrition Information. The Dietary Reference Intakes: The Essential Guide to Nutrient Requirements*.

[9] Szulc P., Chapuy M.-C., Meunier P. J., Delmas P. D. (1996). Serum undercarboxylated osteocalcin is a marker of the risk of hip fracture: a three year follow-up study. *Bone*.

[10] Gajic-Veljanoski O., Bayoumi A. M., Tomlinson G., Khan K., Cheung A. M. (2012). Vitamin K supplementation for the primary prevention of osteoporotic fractures: is it cost-effective and is future research warranted?. *Osteoporosis International*.

[11] Yamaguchi M. (2006). Regulatory mechanism of food factors in bone metabolism and prevention of osteoporosis. *Yakugaku Zasshi*.

[12] Cockayne S., Adamson J., Lanham-New S. (2006). Vitamin K and the prevention of fractures: systematic review and meta-analysis of randomized controlled trials. *Archives of Internal Medicine*.

[13] Shiraki M., Shiraki Y., Aoki C., Miura M. (2000). Vitamin K2 (menatetrenone) effectively prevents fractures and sustains lumbar bone mineral density in osteoporosis. *Journal of Bone and Mineral Research*.

[14] Iwamoto J., Takeda T., Sato Y. (2004). Effects of vitamin K2 on osteoporosis. *Current Pharmaceutical Design*.

[15] Knapen M. H. J., Drummen N. E., Smit E., Vermeer C., Theuwissen E. (2013). Three-year low-dose menaquinone-7 supplementation helps decrease bone loss in healthy postmenopausal women. *Osteoporosis International*.

[16] Booth S. L., Dallal G., Shea M. K., Gundberg C., Peterson J. W., Dawson-Hughes B. (2008). Effect of vitamin K supplementation on bone loss in elderly men and women. *Journal of Clinical Endocrinology and Metabolism*.

[17] Cheung A. M., Tile L., Lee Y. (2008). Vitamin K supplementation in postmenopausal women with osteopenia (ECKO Trial): a randomized controlled trial. *PLoS Medicine*.

[18] Je S. H., Joo N.-S., Choi B.-H. (2011). Vitamin K supplement along with vitamin D and calcium reduced serum concentration of undercarboxylated osteocalcin while increasing bone mineral density in Korean postmenopausal women over sixty-years-old. *Journal of Korean Medical Science*.

[19] Geleijnse J. M., Vermeer C., Grobbee D. E. (2004). Dietary intake of menaquinone is associated with a reduced risk of coronary heart disease: the Rotterdam Study. *J Nutr*.

[20] El Asmar M. S., Naoum J. J., Arbid E. J. (2014). Vitamin K dependent proteins and the role of vitamin K2 in the modulation of vascular calcification: a review. *Oman Medical Journal*.

[21] Gast G., de Roos N., Sluijs I. (2009). A high menaquinone intake reduces the incidence of coronary heart disease. *Nutrition, Metabolism and Cardiovascular Diseases*.

[22] Shea M. K., Booth S. L., Miller M. E. (2013). Association between circulating vitamin K1 and coronary calcium progression in community-dwelling adults: the multi-ethnic study of atherosclerosis. *American Journal of Clinical Nutrition*.

[23] Neogi T., Booth S. L., Zhang Y. Q. (2006). Low vitamin K status is associated with osteoarthritis in the hand and knee. *Arthritis and Rheumatism*.

[24] Misra D., Booth S. L., Tolstykh I. (2013). Vitamin K deficiency is associated with incident knee osteoarthritis. *American Journal of Medicine*.

[25] Neogi T., Felson D. T., Sarno R., Booth S. L. (2008). Vitamin K in hand osteoarthritis: Results from a randomised clinical trial. *Annals of the Rheumatic Diseases*.

[26] Ebina K., Shi K., Hirao M. (2013). Vitamin K2 administration is associated with decreased disease activity in patients with rheumatoid arthritis. *Modern Rheumatology*.

[27] Abdel-Rahman M. S., Alkady E. A. M., Ahmed S. (2015). Menaquinone-7 as a novel pharmacological therapy in the treatment of rheumatoid arthritis: A clinical study. *European Journal of Pharmacology*.

[28] Vermeer C., Soute B., Ulrich M., van de Loo P. (2004). Vitamin K and the Urogenital Tract. *Pathophysiology of Haemostasis and Thrombosis*.

[29] Chen J., Liu J., Zhang Y., Ye Z., Wang S. (2003). Decreased renal vitamin K-dependent gamma-glutamyl carboxylase activity in calcium oxalate calculi patients. *European Urology*.

[30] Sakamoto N., Wakabayashi I., Sakamoto K. (1999). Low vitamin K intake effects on glucose tolerance in rats. *International Journal for Vitamin and Nutrition Research*.

[31] Choi H., An J. H., Kim S. W. (2011). Vitamin K2 supplementation improves insulin sensitivity via osteocalcin metabolism: a placebo-controlled trial. *Diabetes Care*.

[32] Yoshida M., Jacques P. F., Meigs J. B. (2008). Effect of vitamin K supplementation on insulin resistance in older men and women. *Diabetes Care*.

[33] Manna P., Kalita J. (2016). Beneficial role of vitamin K supplementation on insulin sensitivity, glucose metabolism, and the reduced risk of type 2 diabetes: A review. *Nutrition*.

[34] Lamson D. W., Plaza S. M. (2003). The anticancer effects of vitamin K. *Altern Med Rev*.

[35] Ishizuka M., Kubota K., Shimoda M. (2012). Effect of menatetrenone, a vitamin K2 analog, on recurrence of hepatocellular carcinoma after surgical resection: a prospective randomized controlled trial. *Anticancer Research*.

[36] Yoshida T., Miyazawa K., Kasuga I. (2003). Apoptosis induction of vitamin K2 in lung carcinoma cell lines: the possibility of vitamin K2 therapy for lung cancer. *International Journal of Oncology*.

[37] Showalter S. L., Wang Z., Costantino C. L. (2010). Naturally occurring K vitamins inhibit pancreatic cancer cell survival through a caspase-dependent pathway. *Journal of Gastroenterology and Hepatology*.

[38] Nimptsch K., Rohrmann S., Linseisen J. (2008). Dietary intake of vitamin K and risk of prostate cancer in the Heidelberg cohort of the European Prospective Investigation into Cancer and Nutrition (EPIC-Heidelberg). *American Journal of Clinical Nutrition*.

[39] Denisova N. A., Booth S. L. (2005). Vitamin K and sphingolipid metabolism: Evidence to date. *Nutrition Reviews*.

[40] Ferland G. (2012). Vitamin K and the nervous system: An overview of its actions. *Advances in Nutrition*.

[41] Allison A. C. (2001). The possible role of vitamin K deficiency in the pathogenesis of Alzheimer's disease and in augmenting brain damage associated with cardiovascular disease. *Medical Hypotheses*.

[42] Presse N., Shatenstein B., Kergoat M.-J., Ferland G. (2008). Low Vitamin K Intakes in Community-Dwelling Elders at an Early Stage of Alzheimer's Disease. *Journal of the American Dietetic Association*.

[43] Presse N., Belleville S., Gaudreau P. (2013). Vitamin K status and cognitive function in healthy older adults. *Neurobiology of Aging*.

[44] Annweiler C., Ferland G., Barberger-Gateau P., Brangier A., Rolland Y., Beauchet O. (2014). Vitamin K antagonists and cognitive impairment: results from a cross-sectional pilot study among geriatric patients. *The Journals of Gerontology Series A: Biological Sciences and Medical Sciences*.

[45] Pearson D. A. (2007). Bone health and osteoporosis: the role of vitamin K and potential antagonism by anticoagulants. *Nutrition in Clinical Practice*.

[46] Sconce E., Avery P., Wynne H., Kamali F. (2007). Vitamin K supplementation can improve stability of anticoagulation for patients with unexplained variability in response to warfarin. *Blood*.

[47] Leblanc C., Presse N., Lalonde G., Dumas S., Ferland G. (2014). Higher vitamin K intake is associated with better INR control and a decreased need for INR tests in long-term warfarin therapy. *Thrombosis Research*.

[48] Schurgers L. J., Teunissen K. J. F., Hamulyák K., Knapen M. H. J., Vik H., Vermeer C. (2007). Vitamin K-containing dietary supplements: comparison of synthetic vitamin K1 and natto-derived menaquinone-7. *Blood*.

[49] Fusaro M., Tripepi G., Noale M. (2013). Prevalence of vertebral fractures, vascular calcifications, and mortality in warfarin treated hemodialysis patients. *Current Vascular Pharmacology*.

[50] Tagalakis V., Tamim H., Blostein M., Hanley J. A., Kahn S. R. (2013). Risk of prostate cancer death in long-Term users of warfarin: A population-based case-control study. *Cancer Causes and Control*.

[51] Misra D., Zhang Y., Peloquin C., Choi H. K., Kiel D. P., Neogi T. (2014). Incident long-term warfarin use and risk of osteoporotic fractures: Propensity-score matched cohort of elders with new onset atrial fibrillation. *Osteoporosis International*.

[52] Hamidi M. S., Cheung A. M. (2014). Vitamin K and musculoskeletal health in postmenopausal women. *Molecular Nutrition and Food Research*.

[53] Fusaro M., Crepaldi G., Maggi S. (2011). Bleeding, vertebral fractures and vascular calcifications in patients treated with warfarin: hope for lower risks with alternative therapies. *Current Vascular Pharmacology*.

